# Biofilms by bacterial human pathogens: Clinical relevance - development, composition and regulation - therapeutical strategies

**DOI:** 10.15698/mic2021.02.741

**Published:** 2021-02-01

**Authors:** Adina Schulze, Fabian Mitterer, Joao P. Pombo, Stefan Schild

**Affiliations:** 1 Institute of Molecular Biosciences, University of Graz, Humboldtstrasse 50, 8010 Graz, Austria.; 2 BioTechMed Graz, Austria.; 3 Field of Excellence Biohealth – University of Graz, Graz, Austria.; # A.S. and F.M. contributed equally to this work.

**Keywords:** biofilm-associated disease, nosocomial infections, Vibrio cholerae, Pseudomonas aeruginosa, staphylococci, treatment, biofilm

## Abstract

Notably, bacterial biofilm formation is increasingly recognized as a passive virulence factor facilitating many infectious disease processes. In this review we will focus on bacterial biofilms formed by human pathogens and highlight their relevance for diverse diseases. Along biofilm composition and regulation emphasis is laid on the intensively studied biofilms of *Vibrio cholerae, Pseudomonas aeruginosa* and *Staphylococcus spp.*, which are commonly used as biofilm model organisms and therefore contribute to our general understanding of bacterial biofilm (patho-)physiology. Finally, therapeutical intervention strategies targeting biofilms will be discussed.

## INTRODUCTION

Biofilms are communities of microbes embedded in an extracellular matrix that is produced by the microbes themselves. The microbial community may be composed out of one or multiple species, which may be phylogenetically unrelated. Biofilms can either be single or multilayered. The various gradients that exist within biofilm matrices generate micro-niches, which are colonized by microorganisms that have optimized their metabolism for the respective environment. Anaerobic microorganisms, for example, would be found within the deeper layers of the biofilm, but deeper layers of the biofilm are also inhabited by microbial cells that are more sensible to environmental stressors, like hazardous chemical compounds, pH or physical damage. Differentiation of the microbes within the biofilm is aided by the biofilm's role as a mediator of cell-to-cell signaling.

Biofilms have been found to be ubiquitous in almost every environment. They can develop on all non-shedding surfaces in non-sterile liquid or wet environments sticking to both biotic and abiotic surfaces. Biofilms are being produced in the harshest environments, like in hot springs and deep-sea vents, on rocks and soil, the roots and stems of plants, on chitinous surfaces of aquatic animals, but also on many man made surfaces like pipes, the underside of ships, shower hoses etc. Biofilms represent an important element in many food chains in aquatic environment, where they are consumed by invertebrate, which are prey of fish.

**Figure 1:**
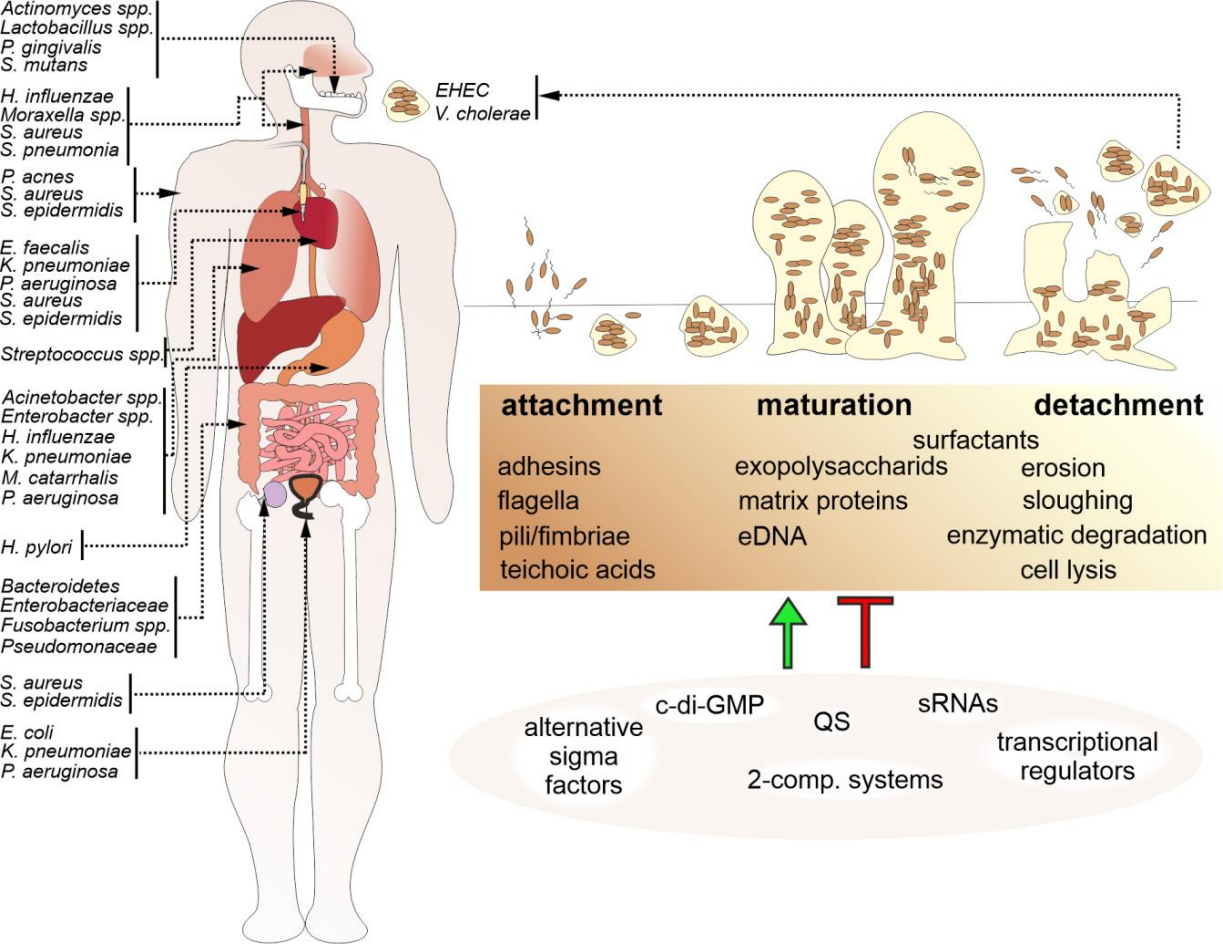
FIGURE 1: Biofilm formation is a common feature among bacterial human pathogens. Bacterial biofilms by human pathogens are found on various tissues of the human body, on medical devices, e.g. catheters or prostheses, and in the environment, representing a reservoir for new infections. A schematic overview indicating representative bacterial species associated with biofilm-related diseases and their occurrence in the body (arrows) is presented on the left. Biofilm formation (upper right) is a multistep process organized in an attachment, maturation and detachment phase. Biofilm formation is controlled and modulated by several factors including bacterial surface molecules, secreted matrix effectors, as well as environmental components and stressors. Thus, it is not surprising that bacterial biofilm regulation (lower right) involves the interplay of several positive and negative regulatory cascades including quorum sensing systems (QS), regulatory small RNAs (sRNAs), alternative sigma factors, two-component systems and second messengers, such as c-di-GMP.

Niels Høiby was amongst the first ones to recognize the relevance of biofilms in disease, which has been supported by increasing evidence since then [1]. Biofilms are involved in a wide variety of microbial infections in the body (**Fig. 1**). The National Institutes of Health (NIH) revealed that among all microbial infections, 60-80% are linked to biofilm formation [2]. Biofilm formation not only occurs on medical devices such as contact lenses, catheters, prostheses, heart valves and pacemakers, but also on a variety of body surfaces, including the skin or mucosal surfaces of the respiratory and digestive tract. Moreover, biofilms formed in the environment are not only a likely survival and persistence stage for facultative pathogens outside the host, but also a relevant reservoir for the initiation of new infections. Several studies have demonstrated that bacteria associated in biofilms exhibit increased resistance to antimicrobial compounds than their individual, planktonic counterparts. Antibiotic resistance in biofilm infections is thought to be caused by a variety of factors, including metabolic alterations in bacteria within the biofilm, decreased penetration of antibiotics due to the extracellular matrix, inactivation of the antibiotic by compounds within the extracellular matrix, inoculum effects related to the very large number of bacteria in the biofilm relative to the available antibiotic molecules and increased exchange of bacterial resistance mechanisms as bacteria reside in close proximity to each other. Bacterial biofilms also facilitate immune evasion, for example by preventing phagocytosis or immune cell modulation and dysfunction via release of bacterial byproducts or toxins. Not surprisingly, hospitals have to deal with diverse nosocomial infections caused by biofilm-forming bacterial pathogens that may severely affect patients suffering from predispositions like immune suppression or pre-existing diseases.

## ENVIRONMENTAL BIOFILMS AND THEIR IMPACT ON TRANSMISSION

Between outbreaks facultative human pathogens may form biofilms outside of the host as a persistence mode. Importantly, biofilm formation can facilitate environmental survival and thereby allows to maintain a high infectious dose even for prolonged inter-epidemic periods. Thus, biofilm communities can represent a reservoir for future infections. Upon infection bacterial cells associated in biofilms are generally better protected against host defense mechanisms than their planktonic counterparts. Thus, biofilms could be a likely form in which opportunistic bacterial pathogens initiate the infection of a human host.

A representative example is *Vibrio cholerae*, the causative agent of the water borne diarrheal disease cholera. *V. cholerae* transits between the aquatic reservoir, where it forms biofilms on chitinous surfaces, and the human host, where it efficiently colonizes the intestinal tract. Importantly, not only intact biofilms, but also *V. cholerae* cells dispersed from a biofilm are more infectious than free-living, planktonic cells in the infant mouse model [3, 4]. These results suggest the existence of factors specifically induced during biofilm formation that facilitate infection by *V. cholerae* even beyond the general idea of being better protected against host-derived antimicrobial factors within a biofilm. The impact of biofilms on transmission of *V. cholerae* is highlighted by the fact that a simple sari cloth filtration of drinking water, effectively removing biofilm-associated bacteria, reduced the number of cholera cases by approximately 50% in an Indian household study [5]. Thus, bacterial clumps or aggregates derived from mature biofilms are a likely form in which clinically relevant *V. cholerae* are taken up by humans, reinforcing the ecological and epidemiological role of biofilms.

Another example is the enterohemorrhagic *Escherichia coli* (EHEC) O104:H4 isolate, which showed increased biofilm formation on fenugreek seeds and caused a severe outbreak in Germany in 2011 with a higher rate of hemolytic-uremic syndrome than any recorded before [6]. Genome-wide sequence analyses revealed that the outbreak EHEC strain had acquired the novel diguanylate cyclase, DgcX, synthesizing the biofilm-promoting second messenger c-di-GMP. Expression levels of DgcX are higher than any other known *E. coli* diguanylate cyclase and it consequently fuels enhanced biofilm formation [7]. One explanation for the unprecedented severity of this EHEC outbreak might be explained by the increased biofilm formation capacity of O104:H4 providing a concentrative infective dose of the pathogen organized in biofilm aggregates.

Environmental biofilms in drinking water systems serve as a reservoir for the respiratory tract pathogen *Legionella pneumophila*, causative agent of Legionnaires disease, and opportunistic pathogens like *Mycobacterium avium*, representing a health risk especially for immunocompromised patients. Especially in shower hoses *Legionella spp.* commonly produces biofilms, which are thought to promote the persistence and chlorine-resistance of the respiratory pathogen [8].

## CHRONIC AND ACUTE DISEASES CAUSED BY BIOFILM FORMING BACTERIA

In contrast to biofilms formed outside of the human, bacterial biofilms can also be key factors for the fitness of pathogenic strains during host colonization. These biofilms can be either associated with medical devices or formed independently from foreign body material via colonization of host tissue, which is mainly observed along chronic infections.

### Medical device-related bacterial biofilms

In clinics, bacterial biofilm formation on foreign body implants, such as catheters (intravascular and urinary), orthopedic inserts as well as dental and breast implants, can result in severe infections. Most infections acquired in a hospital environment (nosocomial diseases) are implant-associated infections and comprise 50–70% of all nosocomial infections [9]. Biofilms on medical devices pose a huge danger due to the high resistance to antibiotics, providing a reservoir of bacteria that can cause constant re-infections and chronic inflammation that can also lead to tissue damage, clogging of devices and general resistance to treatment. Important microorganisms involved in health care associated infections comprise Gram-positive bacteria, e. g. *Staphylococcus aureus, Staphylococcus epidermidis*, and *Enterococcus faecalis* as well as Gram-negative bacteria, such as *E. coli, Klebsiella pneumoniae, Proteus mirabilis*, and *Pseudomonas. aeruginosa* [10].

Roughly 80% of the microorganisms engaged in material-related contaminations are *S. epidermidis* and *S. aureus*, the latter especially in connection with surgical site infections, causing chronic wounds and other issues [11]. Notably, the majority of these isolates exhibit multidrug resistance, posing an immense challenge for therapeutical intervention in clinical practice [11].

Regarding vascular catheters, it has been documented that within the initial seven days after catheterization, extraluminal biofilm by S*. epidermidis, S. aureus, E. faecalis, K. pneumoniae,* and *P. aeruginosa* as well as the fungal pathogen *Candida albicans* considered a significant reason for catheter-related circulation system contaminations. In fact, vascular catheters that had been *in situ* for more than 30 days showed proof of heavy luminal colonization and biofilm development [12].

Along catheter-associated urinary tract infections, also known as CAUTIs, *P. aeruginosa* is one of the main causes in device related bacterial infections. Another dangerous biofilm producer linked to urinary tract infections, also known as UTIs, is *K. pneumoniae*. 63% of *K. pneumoniae* isolates from urine samples of catheterized patients suffering from UTIs were positive for *in vitro* biofilm production [13]. Chronic issues induced by device related infections are often due to the biofilm production enabling a tenacious and persistent colonization. As such, urinary catheters have to be exchanged at least every three months.

Biofilms also play a huge role in ventilator-associated pneumonia that occurs in patients requiring mechanical ventilation breathing machines in hospitals after surgery or various diseases, such as COVID-19. Due to the patients in need of ventilator assisted breathing often suffering from underlying immune or lung problems, ventilator-associated pneumonia can be a life-threatening condition. Ventilator-associated pneumonia has been recorded as pervasive after 48–72 h in patients who have been intubated and are on mechanical ventilation. The increased danger of triggering ventilator-associated pneumonia following intubation with mechanical ventilation is six to 20-fold. Especially endotracheal tubes are often associated with the development of biofilms and the methicillin-resistant *S. aureus*, also known as MRSA, and Gram-negative bacilli, such as, *K. pneumoniae, E. coli, P. aeruginosa,* and *Acinetobacter baumanii* [14]. Not surprisingly, mortality rates of ventilator-associated pneumonia are fundamentally higher than for UTIs and skin diseases [15].

### The respiratory tract

The large mucosal surface makes the respiratory tract a preferred niche for biofilm growth, which can result in chronic inflammation of the mucosal tissue and reduced pulmonary function. For example, the widespread inflammatory disease chronic rhinosinusitis can be linked to presence of bacterial biofilms of the upper respiratory tract. *S. aureus* biofilms have been found on the nasal mucosal surface of 50% of patients [16], but additional causative agents include *Streptococcus pneumoniae, Haemophilus influenzae* and *Moraxella catarrhalis* [17]. The latter two tend to form inter-species biofilms, making treatment even more complicated.

Chronic phenotypes of pharyngitis and laryngitis are frequently associated with biofilm formation. A recent study identified biofilms in 62% patients with chronic laryngitis [18], consisting of pathogens like *S. aureus, H. influenzae, C. albicans, Moraxella nonliquefaciens, Propionibacterium acnes, Neisseria meningitidis*, and *S. pneumoniae* [18]. Substantial biofilm formation might explain the requirement for extended and multiple deployment of antibiotics to treat certain cases of chronic laryngitis.

Bacterial biofilms are also frequently associated with chronic infections of the lower respiratory tract, mainly observed in predisposed patients suffering from abnormal mucociliary clearance and other impaired host defenses, such as cystic fibrosis (CF). Chronic infections of the lung can exacerbate the primary disease and result in destructive inflammation. The altered viscosity, lower sheer and nutrient richness of patient's mucosa seems to promote biofilm formation [19]. While the lower respiratory tract of young patients with CF is prone to infections of *H. influenzae* and *S. aureus*, the main cause for infection in the lungs of adult CF patients is *P. aeruginosa* [20]. If initial colonization is not prevented, *P. aeruginosa* establishes permanently in the lungs and often mucoid mutants are selected that overproduce alginate. The conversion to mucoid strains seems to be driven be thy lung microenvironment and is not observed outside of the human body.

Notably, the extracellular polymers of *P. aeruginosa* biofilms are different for lung and UTIs described above as they contain higher amounts of the exopolysaccharide alginate and extracellular DNA (eDNA) [21, 22]. Alginate protects *P. aeruginosa* against phagocytosis, opsonization, antimicrobial compounds and clearance from the lungs [23]. On the other hand, alginate fuels an immune complex-mediated inflammation via a pronounced antibody response, which is characteristic for a Th2 polarized immune response [24]. Overall, this results in severe lung tissue damage. Notably, *P. aeruginosa* can reside asymptomatically within the human body until biofilm formation has reached a threshold and overwhelms the immune system. Mucoid strains are able to effectively colonize the lungs, stay persistent in the lungs of CF patients and are very difficult to treat. Consequently, biofilm production seems to be the most important virulence factor for *P. aeruginosa* associated with high mortality and morbidity in CF patients.

The exact mechanisms how *P. aeruginosa* biofilms are effectively protected against antibiotics is still a question of ongoing research. In the case of positively charged aminoglycosides, the negatively charged matrix components, e. g. alginate or eDNA, allow only slow diffusion into the biofilm and extend the adaption time for bacteria to mount a stress response [25]. Other antibiotics don't seem to be hindered by the barrier function of the biofilm matrix, but are yet still less effective against *P. aeruginosa* biofilms compared to planktonic bacteria. It is hypothesized that the biofilm provides a privileged environment for drug-tolerant persister cells to survive, which can tolerate antimicrobials for prolonged periods [23].

Exposure of *P. aeruginosa* to hydrogen peroxide or activated polymorphonuclear neutrophils induces a mutation in the *mucA* gene, changing it to the characteristic mucoid phenotype. A Brazilian study revealed that this mutation can be found in 93% of mucoid *P. aeruginosa* isolated from CF patients [26]. In general, *P. aeruginosa* biofilm growth in CF lungs is associated with an increased frequency of mutations, slow growth and adaptation of the bacteria to the conditions in the lungs, and to antibiotic therapy. Thus, *P. aeruginosa* biofilms in CF patients can only be prevented by early aggressive antibiotic prophylaxis or therapy, before the biofilm is fully developed, or they can be treated by chronic suppressive maintenance therapy once the biofilm is already fully developed to extend lung function for several years [27].

Patients with chronic obstructive pulmonary disease (COPD) have a high risk of an acute excerbation triggered by bacterial infections caused by *Pseudomonas, Klebsiella, Acinetobacter, Enterobacter, Moraxella catarrhalis* and mixed infections such as *Pseudomonas* and *Klebsiella* or *Pseudomonas* and *Acinetobacter* [28].

Along these species enhanced biofilm production is often associated with clinical isolates. For example, around 85% of clinical isolates of *K. pneumoniae* exhibit robust biofilm production, which is also associated with multiple drug resistance [29]. Although biofilm production is often described in the context of infections of COPD affected lungs, direct demonstration of biofilm formation in lungs is mostly lacking and verification still remains mostly by indirect means.

### The urogenital tract

A healthy urinary tract is occupied by a diverse natural bacterial flora resulting in relative high acidity by bacterial metabolism and thereby fairly protected from bacterial infections. Thus, main causes of biofilm-associated bacterial infections in the urogenital tract are device-related (see above). However, device-unrelated UTI through smear infection can occur. Notably, biofilm formation capacity of uropathogenic *E. coli* (UPEC) and *S. aureus* isolates was correlated with genitourinary tract infections in several studies [30, 31]. Biofilm producing bacteria can exacerbate infections due to their relatively high antibiotic resistance, which may turn acute infections into chronic or reoccurring infections. For example, about 20% of women with acute cystitis (inflammation of the bladder) suffer from reoccurring UTI mostly caused by bacterial strains with strong biofilm production. Consistently, UPEC strains involved in reoccurring UTIs are better biofilm producers than UPEC strains causing only single episodes [32]. A recent study focusing on UTIs caused by *S. aureus* revealed that 69% of patients' isolates exhibit strong biofilm production, which resulted in increased resistance to nitrofurantoin, tetracycline, erythromycin and ciprofloxacin compared to non-biofilm producing strains [30]. Concordantly, a study focusing on chronic bacterial prostatitis demonstrated that approx. 85% of 150 different bacterial isolates from chronic bacterial prostatitis patients were strong or moderate biofilm producers, including strains like *E. faecalis, Staphylococcus spp., E. coli*, and 20 other Gram-negative rods [33].

### Digestive tract

The digestive tract of the human body is colonized with a vast quantity and diversity of microbes, with the highest concentration in the colon. Already more than 700 different bacterial species reside in the oral cavity of humans [34], which can initiate formation of dental biofilms, also known as dental plaque. The exact composition of the dental biofilm varies not only between different sites in the oral cavity, but also between individuals. Despite this, a core microbiome has been proposed, and includes species of the following genera: *Streptococcus, Veillonella, Granulicatella, Neisseria, Haemophilus, Corynebacterium, Rothia, Actinomyces, Prevotella, Capnocytophaga, Porphyromonas,* and *Fusobacterium* [35]

The dental biofilm can cause diseases in the teeth and their supporting tissues, i.e. dental caries and periodontal diseases. Regular removal of dental plaque is essential, as with increasing biofilm thickness bacteria are better protected against bactericidal activities of the saliva, which can no longer penetrate or reach the whole tooth [36].

Dental caries is characterized by a demineralization of the teeth without concurrent inflammation of surrounding tissues. However, if left untreated it may develop into inflammatory infections, such as pulpitis and apical periodontitis. While especially *Streptococcus mutans, Actinomyces*, and *Lactobacillus spp.* were previously regarded as responsible for caries, the list of caries-associated bacteria now includes species of the genera *Actinomyces, Lactobacillus, Dialister, Eubacterium, Olsenella, Bifidobacterium, Atopobium, Propionibacterium, Scardovia, Abiotrophia, Selenomonas,* and *Veillonella* in addition to carbohydrate-fermenting oral streptococci. Many of them are still not cultivatable in the laboratory. Usually when *S. mutans* colonizes tooth cavities caries follows after six to 24 months [37] The cariogenicity of *S. mutans* is due to the adherence properties of its secreted extracellular polymeric substances (EPSs), production of which is fueled in part by fructose [38].

Periodontal diseases, such as gingivitis and periodontitis are chronic inflammatory diseases of tissue around the teeth. Gingivitis is an inflammation of the gums, frequently observed as a response of the surrounding tissue to bacterial biofilm formation on the teeth. While under healthy conditions the gingival sulcus is colonized with predominantly Gram-positive streptococci at relative low level [39] the microflora can change within a couple of weeks in a complex mixture of mainly anaerobic Gram-positive and -negative bacteria if biofilm formation is not prevented. Prolonged colonization of the oral cavity facilitates further invasion into the mucosal tissue and distribution of bacterial toxins. As a consequence, gingivitis can exacerbate into periodontitis, if no action in intervention of supragingival biofilm formation is taken. The growing biofilm can then extend into the periodontal pocket and manifests as a subgingival biofilm. Biofilms and the ongoing inflammation will gradually result in an opening of the periodontal pockets, disintegration of periodontal fibers and destruction of bones, which will loosen the teeth and finally results in their loss [40]. In contrast to gingivitis, the tissue destruction in periodontitis is irreversible. The subgingival biofilms are dominated by diverse Gram-negative rods like *Prevotella spp., Porphyromonas gingivalis,* and *Fusobacterium nucleatum,* but also include motile bacteria and spirochetes in deeper layers close to the epithelial surface [35].

Notably, the biofilm plaque serves as a constant reservoir of microbes as well as their inflammatory effectors, both of which can spread systematically in the body. Thus, dental biofilm bacteria are also directly and indirectly associated with several other systemic diseases such as cardiovascular diseases, atherosclerosis, infective endocarditis, aspiration pneumonia, diabetes mellitus, preterm birth, and low birth weight babies [41].

The gastric mucosa of approximately 50% of the human population is colonized by *Helicobacter pylori* [42]. Colonization with *H. pylori* is linked to the initiation of peptic ulcer disease, corpus-predominant gastritis, and possibly also esophageal, adenocarcinomas [42]. Organization of *H. pylori in* biofilms has been visualized within the gastric mucosa [43]. One of the best studied virulence factors of *H. pylori* is urease, neutralizing the acidic conditions in the immediate gastric environment cells [44]. Notably, in patients suffering from peptic ulcer disease more than 95% of the mucosal gastric surface was covered by bacterial biofilms in urease-positive biopsies, while less than 2% of the surface was covered in urease-negative biopsies [45]. The importance of *in vivo* biofilm formation by *H. pylori* is also highlighted by a recent study demonstrating that combinatory treatment with antibiotics coupled with the biofilm disrupting compound *N*-acetylcysteine eradicated *H. pylori* in 2/3 of the patients, while a sole antibiotic therapy only cleared the infection in 1/5 of the patients [46].

The residual intestinal mucosa is colonized with an enormous quantity and diversity of bacterial microbiota generally growing as healthy biofilm communities [47]. While defined pathogens cause distinct acute diarrheal diseases, the etiology and link to defined bacterial species for inflammatory bowel disease (IBD), irritable bowel syndrome and colorectal cancer is less clear. However, it is widely accepted that the intestinal microbiota can have beneficial as well as adverse effects on these disease states [48, 49]. For example, in case of ulcerative colitis, a chronic relapsing form of IBD, a variety of biofilm-producing species including *Fusobacterium spp., Shigella spp.* and adhesive *E. coli* have been implicated to promote initiation and maintenance of disease [50]. Similarly, Crohn's disease has been associated with an overall increase of *Enterobacteriaceae, Pseudomonas spp* and *Bacteroidetes*, bacterial groups known to have members with good biofilm forming capabilities [51].

It seems reasonable, that bacterial biofilms can promote chronic colonization of these bacterial groups in gut. Moreover, the relatively high antimicrobial resistance of biofilms would explain the observed intractability of IBD to antibiotic therapy. Finally, biofilm matrix components may potentiate the proinflammatory response, which is a hallmark of IBD. Importance of bacterial biofilms in the pathogenesis of ulcerative colitis and Crohn's disease is indeed suggested by several reports, but we are just at the beginning to understand their impact on IBD and a comprehensive mechanistic understanding is currently lacking [52].

### Skin and wounds

More than 60% of the microbial load on the human skin is composed of diverse biofilm producing bacteria. The predominant floras include *Staphylococcus spp*., *Corynebacterium spp*., and *Propionibacterium spp*. [53]. Biofilm producing skin bacteria cause a number of skin diseases, such as acne vulgaris caused by *P. acnes*, cellulitis, erysipelas and erythema nodosum caused by *Streptococcus pyogenes*, impetigo caused by *S. pyogenes* and *S. aureus*, necrotizing fasciitis caused by *S. pyogenes, Klebsiella* and *Clostridium* amongst others, staphylococcal scaled skin syndrome caused by *S. aureus*, chronic ulcers caused by *Bacteroides, Clostridium* and *Streptococcus,* and finally otitis externa and chronic wounds caused by *P. aeruginosa*. In general, biofilms increase the bacterial fitness against host immune defenses, bacteria, antibiotic therapy and general hygiene treatment. Bacterial biofilms also impact the risk of infection and progression of chronic wounds, as they have been associated with increased wound development and skin infections as well as improper wound healing due to chronic inflammation [54].

Many studies have confirmed that dermal tissues of chronic wounds contain several biofilm-forming bacteria, such as *S. aureus, S. epidermidis, P. aeruginosa, E. coli, Enterobacter spp., E. faecalis,* and *K. pneumoniae*. Almost 88–98% of wound infections have been found to be *S. aureus* positive [55]. *S. aureus* has fibrin receptors and thus can bind to fibrinogen, which can start biofilm formation. This affinity of *S. aureus* to bind to fibronectin, collagen and laminin makes it easy for the pathogen to colonize various host surfaces such as the skin. Patients having *S. aureus* biofilm infections require extended healing times due to delay in re-epithelialization of the infected tissue [56]. This can often exacerbate in patients that suffer from other diseases such as diabetes mellitus, which already damages the patients' tissue. *S. aureus* biofilms are hard to deal with due to their incredible resistance to antibiotic therapy and host immune response, with biofilm production even being promoted by the presence of β-lactam antibiotics and cytokines [57]. Generally, antibiotic resistant *S. aureus* strains, such as MRSA, pose a worldwide problem in clinical medicine. *S. aureus* and *P. aeruginosa* are the two most common causes of chronic wound infections and are frequently co-isolated from the same wound. Chronic wounds don't always only contain chronic infection of a single bacterial strain, but can co-occur with several different biofilm producing strains such as *S. aureus* and *P. aeruginosa*. Analysis of 22 patient samples by using specific peptide nucleic acid and fluorescence *in situ* hybridization revealed that *P. aeruginosa* colonizes the deeper layers in the wound bed, while *S. aureus* was rather found on the wound surface [58]. Recent data indicates that both bacteria benefit from each other in coinfected wounds and synergistically increase antibiotic tolerance [59]. Wounds infected with *P. aeruginosa* are larger in size and require longer healing periods [58].

Emerging data also suggests that biofilm formation is a key colonization factor of the opportunistic pathogen *P. acnes* associated with the inflammatory disease acne vulgaris as well as invasive infections of skin, the cardiovascular system, soft and deep organ tissue and implant associated infections [60]. Most likely biofilm formation in sebaceous follicles results in elevated resistance of *P. acnes* against [61]. Biofilm-like aggregates of *P. acnes* are more frequently observed in skin biopsies of acne vulgaris patients compared to healthy control groups. Moreover, recent data suggests that biofilm formation by *P. acnes* is phylotype-dependent and isolates derived from invasive infections are associated with better biofilm production compared to healthy skin isolates [62, 63].

### Biofilms associated with invasive disease

Invasive microbial infections occur at parts of the body that are generally considered germ free, e. g. the blood or other internal fluids and internal body sites such as the brain or the heart. Even though the infection routes can vary, some invasive microbial infections correlate with the ability of the responsible microbes to form biofilms. Well known biofilm associated invasive microbial diseases include endocarditis caused by *Streptococcus*, osteomyelitis mainly caused by *S. aureus*, otitis media caused by *S. pneumonia* and *H. influenzae* and meningitis caused by *A. baumannii* and *H. influenzae*.

Although bacterial endocarditis is mostly linked with heart implants, it can also occur through microbes reaching the heart either through wounds or in some cases through the bloodstream during the course of an invasive infection [64]. Microbes like *Streptococcus spp.* have fibronectin receptors facilitating biofilm formation on different tissues at various sites of injury, which can cause tissue damage of the valves and is especially detrimental in the case of endocarditis [65]. Open fractures, beside posing immediate danger to health, can also lead to chronic infections such as the bone disease osteomyelitis. *S. aureus* is predominantly present as a causative agent in cases of invasive osteomyelitis [66]. *S. aureus* has fibrin receptors and thus can bind to fibrinogen present in the bone matrix and can start biofilm formation. This affinity of *S. aureus* to bind to fibronectin, collagen and laminin makes it easy for the pathogen to colonize the bone by forming a biofilm [67]. One of the more predominant invasive diseases is otitis media, an infection of the inner ear. *S. pneumonia* and *H. influenzae* both cause otitis media, with more and more biofilm forming serotypes emerging as antibiotic treatment increases pointing to an important role of biofilms as protective factors in those cases [68].

Clinical isolates of invasive non-typeable *H. influenzae* and *A. baumanii* from bacterial meningitis patients, demonstrate higher biofilm production compared to isolates of these species, derived from carriers, chronic disease or respiratory tract infections [69, 70], which emphasizes the impact of biofilm formation for these pathogens to cause invasive diseases. Although biofilm formation is not directly linked to bacterial meningitis caused by *Neisseria meningitidis*, biofilm production is an important mucosal survival and persistence factor for the bacterium. Approximately 30% of carriage isolates are strong biofilm producers, a far greater percentage as observed for acute disease isolates. This suggests that biofilms might be important for the chronic carriage of the bacterium, which provides a reservoir for invasive meningococcal disease [71].

## BACTERIAL BIOFILM FORMATION AND COMPOSITION

Based on the contribution of bacterial biofilms to bacterial infections, bacterial biofilm development and composition became a focal point of interest within the scientific community. Bacterial biofilm formation is a multistep process (**Fig. 1**): In general, initial surface attachment of planktonic bacteria is reinforced via adhesive surface appendages or proteins. Upon irreversible attachment and microcolony formation bacteria induce factors for production and secretion of extracellular matrix components, which results in the formation of a three-dimensional biofilm architecture. Finally, a mature biofilm requires dispersal to avoid harmful overgrowth, nutrient limitation and accumulation of metabolic waste products. Thus, some bacteria will detach from the mature biofilm to resume a planktonic lifestyle.

Formation and maintenance of biofilms require extracellular matrix components, which are responsible for surface adhesion, cell binding and preserving the biofilm architecture (**Fig. 1**, **Table 1**). Not only is there a vast diversity of the microbial community, but also the extracellular matrix shows species-specific variability. The EPS secreted by the constituent population of the biofilm is the major component of bacterial biofilms. The EPS mainly consist of polysaccharides, but may also contain other biomolecules like proteins, nucleic acids, glycopeptides, lipids, lipopolysaccharides as well as sequestered metals.

**TABLE 1.** **Overview of factors involved in the different stages of biofilm formation for the bacterial pathogens *V. cholerae, P. aeruginosa, S. aureus* and *S. epidermidis* discussed in this article.** For details we kindly refer to the text (see chapter “Bacterial biofilm formation and composition”).

**stage in biofilm formation**	**bacterial pathogen**		
	***V. cholerae***	***P. aeruginosa***	***S. aureus/epidermidis***
**attachment**	flagella motility, type IV pili, adhesins and chitin-binding factors (e.g. GbpA, ChiRP, FrhA, CraA)	flagella/twiching motility, type IV pili, Cup fimbrial adhesins and lectins	hydrophobic surface, teichoic acids, adhesins (e.g. Atl, Bap, MSCRAMMs, SERAMs)
**maturation**	exopolysaccharide (VPS), eDNA, proteinaceous factors (RbmA, RbmC, Bap1), lipids	exopolysaccharide (alginate, Psl, Pel), eDNA, proteinaceous factors (e.g. CdrA, LecA/B), rhamnolipids	exopolysaccharide (PIA), eDNA, proteinaceous factors [e.g. SasG, Aap, and other adhesins (see above)], teichoic acids
**detachment**	nucleases (Dns and Xds), proteases, predicted sugar lyase (RbmD)	Alginate lyase, rhamnolipids, cell lysis	exoproteases (e.g. SspA/Esp, SspN/SepA, SplA-F, ScpA)

Many bacterial species are forming biofilms helping them to persist within the environment, protecting them against the host's immune system and therefore promoting infection and the development of disease symptoms. Here, the focus is laid on *V. cholerae, P. aeruginosa, S. aureus* and *S. epidermidis* due to their overlapping coverage of the mentioned biofilm functions (**Table 1**). *V. cholerae* biofilms formed in the aquatic ecosystem not only facilitate environmental persistence, but also impact transmission of the disease [72]. *P. aeruginosa* biofilms are found on medical devices as well as in the respiratory tract, i.e. in the lungs of CF patients [73]. Finally, biofilms of *S. aureus* and *S. epidermidis* are frequently associated with infections derived from indwelling medical devices and chronic wounds [74, 75]. The selected candidates are well characterized biofilm producers as well as genetically modifiable, allowing deeper phenotypical analyses by the implementation of loss-/gain-of-function constructions.

### Attachment

Bacterial adhesion on surfaces consists of reversible and irreversible stages and involves numerous factors, ranging from flagella, pili, fimbriae, lipopolysaccharides, lipoproteins, membrane proteins, adhesins, and eDNA.

The importance of flagella-mediated motility for initial attachment has been reported for several pathogens, including *V. cholerae* and *P. aeruginosa* [76, 77]. *V. cholerae* uses its single, polar, Na^+^-driven flagellum to swim near the surface [78]. In close proximity to the surface hydrodynamic forces acting on the flagellum and cell body re-direct flagellar rotation into a clockwise direction resulting in circular swimming behavior [79]. Movement of *V. cholerae* becomes more restricted upon tethering to the surface by their flagella [79]. An elegant microscopical study by Utada and coworkers identified two motility modes named “roaming“ and “orbiting” [80]. Besides flagellar motility these motion types require the mannose sensitive hemagglutinin type IV pili (MSHA) of *V. cholerae* promoting mechano-chemical attachment to surfaces. Weak interactions between the surface and MSHA enable bacteria to pass over the surface by long directional movements with only small curvatures, which define the “roaming mode”. In contrast, the “orbiting mode” results from stronger interactions between the surface and MSHA visualized by tight, repetitive movements with near-circular orbits with high curvatures. More and more MSHA-surface interactions may tether orbiting cells tighter to the surface. Eventually, bacteria attach irreversibly to the surface and initiate production and secretion of the *Vibrio* exopolysaccharide (VPS) and biofilm matrix proteins resulting in microcolony formation followed by biofilm maturation (see below “Three-dimensional biofilm formation and maturation”). Notably, non-motile mutants lacking the major flagellin subunit FlaA are still capable of forming biofilms, but aggregate first in liquid culture before the clumps immobilize on surfaces resulting in altered biofilm architecture [79]. Moreover, *flaA* mutants show increased VPS production, which suggests that loss of the flagellum could induce biofilm formation [79]. Mutations in the flagellar motor complex negate the VPS overproduction of *flaA* mutants, indicating that the flagellar motor could act as a mechano-sensor involved in the transition to the irreversible attachment state and initiation of matrix production [81].

It should be emphasized that environmental biofilm formation of *V. cholerae* in aquatic reservoirs occurs on chitinous surfaces, consisting of β-1→4 linked *N*-acetyl-D-glucosamine (GlcNAc) [82]. Several factors promoting attachment to chitin have been reported. For example, the GlcNAc-binding protein GbpA, which seems quite specific for GlcNAc-oligosaccharides, the chitin-regulated type IV pili ChiRP promoting competitive attachment to chitinous surfaces, and the MSHA pili, which generally facilitates adhesion to abiotic and chitinous surfaces, e.g. borosilicate, zooplankton and crab shells [83]. Moreover, the flagellum-regulated hemagglutinin FrhA and c-di-GMP-regulated adhesin A (CraA) promote attachment and initial biofilm formation on chitin [84]. Thus, it is likely that these factors play more crucial roles for biofilm formation in the natural environment than what is currently suggested by laboratory studies mainly focusing on plastic material.

Similar to *V. cholerae, P. aeruginosa* is thought to get into close proximity to the surface via flagella-mediated motility. Non-flagellated mutants show reduced attachment especially under glucose- or amino acid-rich conditions [76, 85]. However, in contrast to *V. cholerae, P. aeruginosa* reversibly attaches to surfaces in an upright (vertical) position and moves along random trajectories in “walking” mode using twitching motility mediated by type IV pili [86]. Mutants with a defective type IV pilus form aberrant biofilms [76]. Upon horizontal orientation to the surface, attachment transits into an irreversible state, but bacterial cells are still active for two-dimensional movement via twitching motility resulting in the organization of microcolonies. Comprehensive studies by the Tolker-Nielsen group suggest that *P. aeruginosa* also uses twitching motility for climbing up microcolonies formed by a subpopulation of non-motile cells to form the typical mushroom-like architecture of a mature biofilm [87]. *P. aeruginosa* recognizes surface attachment via the WspA protein, the membrane-bound receptor protein of the Wsp chemosensory signal transduction system that activates c-di-GMP synthesis upon surface contact [88]. As it will become evident below (see chapter “Regulation”) the second messenger c-di-GMP is a central signal involved in biofilm regulation. In *P. aeruginosa* activation of the Wsp system and high c-di-GMP levels act positively on the production of CdrA (cyclic diguanylate-regulated two-partner secretion partner A) adhesin and Cup fimbrial adhesins, which promote surface adherence, as well as the exopolysaccharides Psl, Pel, and alginate, which are structural parts of the biofilm matrix [89–91].

Regarding biofilm formation of non-motile bacteria, the best studied representatives are probably *S. aureus* and *S. epidermidis*. In absence of a flagellum, adherence to hydrophobic surfaces is facilitated by the overall hydrophobic character of the bacterial envelope [74]. Furthermore, attachment to abiotic surfaces via hydrophilic and ionic interactions is promoted by defined surface factors, including wall teichoic acids, the major autolysin AtlE of *S. epidermidis*, its *S. aureus* homologue Atl, and the surface protein Bap of *S. aureus*, respectively [92, 93]. For example, *atlE* mutants in *S. epidermidis* exhibit a less hydrophilic surface and reduced biofilm formation capacity on polystyrene [94]. *S. aureus dtlA* mutants lack an amino acid substitution in the wall teichoic acids, which increases their negative charge and thereby reduces initial attachment to hydrophobic glass or plastic surfaces [92].

Indwelling devices are rapidly surrounded by host tissue and coated with a host-derived matrix. To initiate biofilm formation, various staphylococcal surface factors not only adhere to host cell surfaces, but also bind extracellular host matrix components, e.g. fibronectin, fibrinogen, vitronectin, thrombospondin, bone sialoprotein, elastin, and collagen [92]. Aside of the above-mentioned wall teichoic acids, autolysins and Bap, these largely comprise the covalently-linked microbial surface components recognizing adhesive matrix molecules (MSCRAMMs) and the non-covalently surface-associated secretable expanded repertoire adhesive molecules (SERAMs). MSCRAMMs contain a conserved domain organization including an N-terminal signal peptide, an outwardly exposed ligand-binding domain with directly repeated sequences, a hydrophobic membrane-spanning region, a C-terminal LPXTG motif required for cell wall anchorage, and a positively charged tail [95]. Cell wall anchorage is predominantly mediated by the SrtA sortase, a membrane-bound transpeptidase covalently linking the protein via the carboxyl group of threonine in the LPXTG motif to the amino group of the peptidoglycan [96]. Due to its conserved role in anchoring virulence factors to the cell wall, SrtA is suggested as a target for anti-virulence drug development against staphylococci, enterococci and streptococci [97]. While *S. aureus* isolates encode for more than 20 MSCRAMMs, there are currently only twelve identified in *S. epidermidis*. Representative examples include the fibronectin-binding proteins FnbPA and FnBPB as well as the fibrinogen-binding proteins ClfA and ClfB of *S. aureus* or the accumulation-associated protein Aap and Bhp of *S. epidermidis*, which are highly homologous to SasG and Bap in *S. aureus*. Attachment flexibility and diversity is ensured as one MSCRAMM can bind several host factors and MSCRAMMs exhibit overlapping binding capacities. Not surprisingly, identification of the individual binding spectra of MSCRAMMs is still ongoing. SERAMs are a loosely defined group of secreted proteins, which bind back to bacterial surface by so far uncharacterized mechanism(s) and have relaxed binding specificity to host matrix factors [98]. Representative examples include the extracellular adherence protein Eap (also known as Map or P70) and the extracellular matrix and plasma binding protein Emb of *S. aureus*, which are absent in *S. epidermidis* [98]. Notably, *S. epidermidis* encodes for the membrane-spanning giant 1.1 mDa fibronectin-binding protein Embp, while Ebh represents the homologue in *S. aureus* [99, 100]. The current knowledge on staphylococcal adhesins was recently reviewed in detail by Heilmann *et al.*, which we suggest for further reading [92].

### Three-dimensional biofilm formation and maturation

Upon surface attachment bacteria alter their expression profile from a planktonic to a sessile lifestyle highlighted by the upregulation of components required for the biofilm matrix formation. The exact biofilm matrix composition differs between species, but generally includes a blend of various secreted biomolecules, such as polysaccharides, eDNA, proteins, lipids, and teichoic acids.

#### Exopolysaccharides

In many bacteria the development of a mature biofilm is associated with the production of exopolysaccharides, which are frequently the major component of the biofilm matrix.

For example, the VPS constitutes up to 50% of the mature *Vibrio* biofilm matrix and is required for the development of a three-dimensional biofilm [101, 102]. It is a polymer with a major repeating unit of 1→4 linked α-L-GulpNAcAGly3OAc, β-D-glucose, α-D-glucose and α-D-galactose, with α-l-GulpNAcAGly being an amide between C-6 of 2-acetamido-2-deoxy-α-l-gulopyranosyluronic acid and an amino group of glycine, OAc being an *O*-acetylation and NAc being a *N*-acetylation [103]. Replacement of α-D-Glc by an α-D-GlcNAc in approximately 20% of the repeating units increases diversity [103]. The two nearby chromosomal loci *vps*-I and *vps*-II encode proteins for VPS biosynthesis and export, which are activated shortly after surface attachment [104, 105]. Notably, the *vps*-I and *vps*-II gene clusters are separated by the *rbmA-E* operon [106, 107], encoding for the two matrix proteins RbmA and RbmC (see below).

*P. aeruginosa* produces three different types of exopolysaccharides, i.e. alginate, Psl (polysaccharide synthesis locus) and Pel (pellicle) [108]. Alginate is an acetylated polymer of β-1,4-linked D-mannuronate and L-guluronate, which is synthesized by enzymes encoded by the *algACD* gene cluster [109]. It is not only the most important structural component of *P. aeruginosa* biofilms, but also acts as a barrier for antimicrobial compounds and facilitates immune evasion, thereby contributing to *in vivo* persistence during lung colonization [110, 111]. Biofilm formation of *P. aeruginosa* independent of alginate production highlights the existence of other polysaccharide matrix components, e.g. Psl and Pel [112, 113]. While Pel is present in most *P. aeruginosa* strains, Psl is not wide-spread and is only produced by few *P. aeruginosa* strains, most notably by strain PAO1, but not PA14. Moreover, while Psl is found mainly at the outer surface of microcolonies, Pel is mainly located at the stem of the mushroom structure [114, 115]. The polysaccharide synthesis locus (*psl*) harbors 15 genes involved in biosynthesis of the extracellular sugar polymer Psl, containing D-mannose, D-glucose and L-rhamnose [116]. Psl can be found in a larger cell-associated form and in a smaller soluble form. Especially, the smaller variant is thought to facilitate intercellular interactions and cell-surface attachment, but the exact mechanism yielding in the smaller variant is currently unclear [117]. Psl not only supports adherence during initial biofilm stages, but also contributes to the structural stability of mature biofilms. In doing so Psl interacts with other abundant biofilm matrix components including the matrix protein CdrA and eDNA [89, 118]. Moreover, *P. aeruginosa* deposits a chemtrail of Psl as it moves on a surface, which guides subsequent cells to microcolony formation [119]. By exploiting the released DNA from the host's neutrophil extracellular traps, originally a defense system against pathogens, the eDNA-Psl interaction acts as biofilm scaffold and facilitates survival of *P. aeruginosa* during lung colonization [118]. Similarly, the Pel polysaccharide can also bind to eDNA due to its cationic amino sugars, which might explain why Pel can partially compensate a lack of Psl in *P. aeruginosa* biofilms [120]. Mutant strains lacking Pel are more susceptible to aminoglycoside antibiotics either because Pel binds aminoglycosides to reduce their activity or blocks their penetration into the biofilm [121]. The *pel* locus comprises a seven gene operon encoding for proteins with predicted functions for biosynthesis of the glucose-rich polysaccharide, but the exact chemical composition of Pel remains to be elucidated [120].

The major biofilm exopolysaccharide class in staphylococci is the polysaccharide intercellular adhesin (PIA) or, according to its chemical composition, a polymer of β1→6 linked N-acetylglucosamines (PNAG), respectively [122]. It is considered to be the most important intercellular adhesin of staphylococci and is crucial for biofilm formation and virulence in *S. epidermidis* [123, 124]. PIA is synthesized by the proteins expressed from the *icaADBC* (intercellular adhesion) operon [124]. The N-acetylglucosamine transferase IcaA, together with IcaD, synthesizes an N-acetyl-glucosamine oligomer [125]. Chain growth is dependent on IcaC, which is suggested to act as PIA exporter. PIA is partially deacetylated on the bacterial surface by the PIA deacetylase IcaB [126]. This step is crucial for PIA retention and thus for the various functions PIA fulfills, not only biofilm formation, but resistance to antimicrobial peptides and neutrophil phagocytosis [126]. Moreover, partial loss of the N-acetyl groups after secretion results in a cationic character facilitating electrostatic interactions with other extracellular molecules and adhesive properties of the biofilm matrix [126].

#### eDNA

It is becoming increasingly evident that eDNA is a polymeric matrix component of many bacterial biofilms and most likely originates from cell lysis [127]. The highly polymeric and anionic features of DNA allow cell-to-cell interactions via surface molecules in the matrix network [128]. Importantly, several bacteria secrete nucleases to degrade eDNA, which makes it a rather flexible structural component and enables bacteria to adapt to environmental changes via eDNA modulation.

For example, in *V. cholerae* biofilms eDNA levels are controlled by the extracellular endonuclease Dns and the exonuclease Xds, which is important for the development of a typical sponge-like biofilm architecture and detachment from mature biofilms [4]. Similar observations have been reported for *P. aeruginosa* with its secreted EndA nuclease, and for *S. aureus* releasing two thermostable nucleases Nuc1 and Nuc2 [129–131]. Due to extracellular nucleases and respective nucleotide uptake systems, eDNA can also serve as a carbon, nitrogen and phosphate source [132–134]. Not surprisingly, phosphate starvation activates nucleases in *V. cholerae* resulting in eDNA degradation and biofilm dispersion [132]. Indeed, this perception is confirmed by results showing that phosphate limitation negatively impacts biofilm formation in *V. cholerae* [135, 136]. Besides its contribution to the biofilm architecture, eDNA is also a major proinflammatory factor of *P. aeruginosa* biofilms, limits penetration of antimicrobial compounds and allows horizontal gene transfer [25, 133, 137].

#### Matrix proteins

Another important component of the bacterial biofilm matrix are proteins. Most proteins studied in the context of biofilm matrix contribute to the adhesive properties, stability and morphology of the biofilm. However, it should be noted that some proteins associated with biofilms exhibit enzymatic properties, e.g. sugar hydrolases, proteases and the above-mentioned nucleases, which actively degrade and modulate other matrix components resulting in biofilm reorganization and dispersal [4, 126, 129–131, 138–142].

In *V. cholerae* three major biofilm matrix proteins with predicted carbohydrate-binding domains have been identified, i.e. Bap1 (Biofilm-associated protein 1) as well as RbmA and RbmC (rugosity and biofilm structure modulator A and C) [106]. Importantly, they exhibit individual spatiotemporal expression profiles and consequently fulfill different roles in biofilm formation, which was comprehensively characterized by an elegant microscopical study by Berk and coworkers [104]. The 26 kDa RbmA appears first on the cell surface after cells have attached to the surface and VPS production was initiated [104]. At later stages RbmA can be found on cell surfaces throughout the entire mature biofilm [104]. RbmA exhibits binding specificity to sugars including sialic acid derivates, which can be found in lipopolysaccharides as well as to galactose, which is a component of VPS [105, 143]. This suggests that surface-located RbmA can act mainly as a scaffold protein mediating intercellular and cell-matrix interactions. Along initial biofilm formation, RbmA secretion is followed by the 75 kDa Bap1, predominantly at sites were cells have contact with the surface or other bacteria. Even in mature biofilms, Bap1 is mainly found at the bottom of the biofilm with highest concentrations close to the founder cells, suggesting that it is predominantly secreted by these early biofilm members. Thus, anchoring the biofilm to the surface seems an important and unique feature of Bap1. Moreover, Bap1 was shown to bind outer membrane vesicles via the porin OmpT [144], which confers resistance to antimicrobial peptides. However, the impact of this interaction on biofilm formation is currently unknown. The last matrix protein to appear on discrete sites of the cell surface is RbmC, with a molecular weight of 104 kDa. Bap1 and RbmC share 47% similarity on the protein level and have several common domains. For example, both matrix proteins contain four *Vibrio-Colwellia-Bradyrhizobium-Shewanella* repeats (VCBS) forming two VCBS regions implicated to aid in cell adhesion [107, 145, 146]. Bap1 also contains four and RbmC two FG-GAP repeats, which are found in the eukaryotic integrin α-chain important for attachment to the extracellular matrix [145, 147, 148]. Furthermore, Bap1 has one jacalin-like lectin domain with binding specificity to galactose, mannose and glucose, while RbmC has two such domains [105, 149]. These sugars are also present in the *Vibrio* exopolysaccharide matrix suggesting interactions between VPS and both proteins in the *Vibrio* biofilm [105]. Indeed, Bap1 and RbmC can partially complement each other, although they are not functionally redundant [107]. Both proteins form flexible envelopes around the cells in the biofilm, but only Bap1 remains at high concentrations on the basis of the biofilm, while RbmC seems rather important for VPS interactions throughout the biofilm [104].

Notably, export of the three matrix proteins, RbmA, Bap1 and RbmC, as well as of the chitin-binding protein GbpA relies on the type II secretion system, which is also responsible for cholera toxin secretion during intestinal colonization [150, 151]. This highlights the importance of this secretion machinery for *V. cholerae* physiology. Type II secretion requires proper protein folding in the periplasm under assistance of chaperones before the machinery recognizes the folded substrates for further translocation across the outer membrane. Interestingly, a recent study indicated that O-glycosylation of periplasmic chaperones impacts the type II-dependent secretion of several proteins, including RbmA, and consequently alters biofilm formation [152].

*P. aeruginosa* biofilms also harbor several matrix proteins, including the Psl-binding protein CdrA as well as the lectins LecA and LecB (also known as PA-IL and PA-IIL) [89, 153]. The matrix protein CdrA is expressed in *P. aeruginosa* biofilms in response to high levels of 3,5,-cyclic diguanylate (c-di-GMP) and binds to the exopolysaccharide Psl, most likely via mannose residues [89]. CdrA contains several potential binding domains, including a carbohydrate-dependent activity domain, a glycine-rich sugar-binding domain and an arginine-glycine-aspartate motif that may facilitate adhesion to integrin [89]. Due to its Psl interaction CdrA mediates cell autoaggregation reinforcing biofilm integrity and development on abiotic surfaces [89]. Notably, CdrA exists in two forms, a 220 kDa cell-associated version and a soluble 150 kDa processed variant, which misses approximately 45 kDa on the N-terminal end and 35 kDa on the C-terminal end, respectively [89]. Functional differences between the two variants remain to be elucidated.

In addition to their cytotoxic activity during lung infection, LecA and LecB also contribute to biofilm formation [154–157]. LecA is required for biofilm formation on abiotic surfaces, such as plastic or stainless steel, and shows high binding affinity to hydrophobic galactosides, but also binds sugars like *N*-acetyl-D-galactosamine and glucose [157–159]. LecA is a tetrameric protein that consists of four 12.8 kDa subunits [160]. Each monomer contains a calcium-dependent ligand-binding site for galactose as well as an additional independent binding site for glucose [159]. However, it is not yet clear whether LecA interacts with Psl, Pel or both. In contrast, LecB is rather required for biofilm formation on glass surfaces and binds to a number of monosaccharides, with high specificity to L-fucose [154]. Nevertheless, LecB readily interacts with mannose and galactose residues of Psl [161]. Similar to LecA, LecB is a tetramer assembled out of four 11.7 kDa subunits with ligand binding pocket stabilized by two calcium ions [162]. LecB is localized to the outer membrane with the outer membrane porin OprF being an essential ligand for its membrane association [163].

Several staphylococcal surface proteins have been attributed adhesive properties along biofilm formation. Along the ones already mentioned in the attachment section above, cell wall-anchored SasG of *S. aureus* and its homologue Aap in *S. epidermidis* exhibit self-polymerizing activity facilitating zinc-dependent intercellular interactions [164]. Interestingly, SasG and Aap can also interact with each other allowing interspecies biofilm formation [165]. Impact on pathogenesis in a mouse catheter implant infection model was demonstrated for the surface proteins Bap and Spa in *S. aureus* [166, 167]. Members of the Bap protein family are large proteins found in the biofilm matrix of several Gram negative and positive bacteria, including *S. aureus* and *S. epidermidis* [166, 168]. The 239 kDa Bap of *S. aureus* exhibits four regions (A-D) that contain repeats as well as a N-terminal putative Sec-dependent signal sequence. Region A consists of two 32 amino acid repeats followed by region B without repeats [166]. Interspecies biofilm formation might be mediated by heterodimerization of Bap orthologs via a putative dimerization domain located in regions A and B [169]. The central region C contains thirteen 86 amino acid repeats, which are predicted to fold in a β-sandwich and mediate adhesion. Finally, region D contains three 18 amino acid repeats and the cell-wall anchoring LPxTG motif at the C-terminus [166].

#### Lipids, surfactants and teichoic acid

Lipids and biosurfactants are also present in the extracellular matrix, e.g. in *V. cholerae* it can amount to 33% of the entire biofilm extracellular matrix [170]. Especially biosurfactants are important for bacterial attachment and dispersal from oil droplets. Generated by microorganisms at the air-water interface, they influence surface tension and gas exchange between estuarine waters and the atmosphere [171]. Rhamnolipids, which act as surfactants, can be found in the extracellular matrix of *P. aeruginosa* [172]. The quorum sensing (QS)-controlled *rhlA* gene encodes for a rhamnosyltransferase that is exclusively involved in rhamnolipid biosynthesis [173]. Rhamnolipids play a crucial role in shaping the biofilm architecture by facilitating surface-associated bacterial migration, the formation of mushroom-shaped structures and keeping the water channels of the biofilm open during matrix maturation [172].

In staphylococcal biofilms, teichoic acids are crucial for adhesion, biofilm formation and host colonization [174, 175]. Two different forms exist: wall teichoic acids, consisting of alternating phosphate and ribitol, are covalently linked to peptidoglycan in the cell wall, while lipoteichoic acids, exhibiting alternating phosphate and glycerol, are surface-anchored via a lipid moiety intercalating with the cytoplasmic membrane [92]. The high charge of teichoic acids is critical for *S. aureus* biofilm formation on abiotic surfaces. Wall teichoic acids lacking D-alanine, and thus increasing the net negative charge, decrease the ability of the microorganism to form *in vitro* biofilms on plastic surfaces [174]. In *S. epidermidis* wall teichoic acids induce adhesion to immobilized fibronectin [176].

### Detachment/dispersal

Finally, a vital biofilm community can only persist when a certain population of bacterial cells are allowed to detach from the mature biofilm community leaving a favorable environment for the remaining residents. Dispersed bacteria can either find a new substratum to attach and initiate biofilm formation or transit into a planktonic lifestyle to explore other niches. Biofilm dispersion ranges from continuous detachment of single cells, also known as erosion, to rapid release of multicellular clumps of the biofilm community, also called sloughing. Dispersal can either be an active mechanism or passively mediated by physical stressors such as shear forces. Active detachment relies on differential gene expression triggered by diverse environmental cues like temperature and pH shifts, nitric oxide, starvation, oxygen deprivation, and other stressors. During dispersal genes involved in cell motility and biofilm matrix degradation are generally induced, while attachment and EPS production genes are repressed [177].

*V. cholerae* biofilms grown on chitinous surfaces disperse within minutes upon removal of Ca^2+^, highlighting the importance of the environmental conditions for biofilm development [178]. Recently, Singh *et al.* observed that mature biofilms in flow cells disintegrated rapidly upon stopping the flow, which results in nutrient depletion as well as accumulation of QS autoinducers [179]. These changes result in altered gene expression mainly mediated via RpoS, an alternative sigma factor rising upon nutrient limitation and HapR, the master regulator of QS with increasing cellular levels upon high cell density [180, 181]. Indeed, mature biofilms with a critical size beyond approximately 18 µm show high levels of RpoS and HapR, both required to initiate dispersal [179, 180]. Furthermore, the extracellular nucleases Dns and Xds have been demonstrated to be essential for detachment [4]. Notably, biofilm clumps of the *xds/dns* double mutant were impaired for *in vivo* colonization, while wild type (WT) biofilm clumps outcompeted their planktonic counterparts [4]. This strengthens the current dogma that biofilm-derived aggregates are a likely form in which *V. cholerae* is ingested by the host, but also highlights the importance of biofilm dispersal in the gut to achieve full colonization fitness. Finally, RbmD, encoding a putative polysaccharide lyase, has been hypothesized to have a role in VPS degradation resulting in detachment as a *rbmD* mutant shows increased biofilm formation [107]. A decrease of the second messenger c-di-GMP liberates the LapG protease from the c-di-GMP receptor LapD, which results in proteolytic cleavage of the surface adhesins CraA and FrhA, promoting biofilm detachment [84].

Low c-di-GMP levels is also considered a signal for biofilm dispersion in *P. aeruginosa*, although the exact mechanism remains to be elucidated. Alginate lyase induction in *P. aeruginosa* resulted in a three-fold reduction of the exopolysaccharide alginate and increased the number of detached cells by nine to 16-fold [182]. Furthermore, *P. aeruginosa* biofilm dispersal is influenced by carbon availability [183]. Biofilms grown on glutamate medium induced dispersal upon excessive carbon availability [183]. The extent of released cells correlated with increased expression of flagella and downregulation of twitching motility. Indeed, flagellated subpopulations leaving *P. aeruginosa* biofilms have been described [184]. Although rhamnolipids are important surfactants along *P. aeruginosa* biofilm development, increased levels can result in bacterial dispersal [184, 185]. Cell lysis may also play an important role in biofilm dispersal. In twelve-day old *P. aeruginosa* biofilms dead cells in the center mount up to 50%, which could be partially attributed to prophage activation [186].

Secretion of exoproteases seems to be the main detachment strategy of *S. aureus* and *S. epidermidis*. *S. aureus* secrets seven serine proteases (SspA and SplA-F), two cysteine proteases (SspB and ScpA) and one metalloprotease (Aur) [187]. SspA degrades the adhesins FnBPs and Bap and aureolysin degrades ClfB and Bap mediating biofilm dispersal [188]. *S. epidermidis* encodes at least three secreted proteases: a homologue of the cysteine protease SspB, the SepA metalloprotease and Esp, a homologue of the serine protease SspA [189]. SspB and ScpA, also called staphopains, are shown to disrupt the biofilm matrix, however, the target proteins are yet to be characterized [190]. Proteases are induced by the *S. aureus* QS-system *agr*, which is activated upon an autoinducing peptide (AIP) [191]. In addition, non-native proteases are likely to impact *S. aureus* biofilm development as most proteases seem to have rather relaxed target specificity [192]. For example, the serine protease Esp of *S. epidermidis* is able to cleave an array of *S. aureus* biofilm proteins, including Eap, FnBPA and Atl and consequently disperses *S. aureus* biofilms [193, 194]. Notably, *S. aureus* and *S. epidermidis* lack enzymes degrading the exopolysaccharide PIA, although such effectors exist in nature. For example, the *Actinobacillus actinomycetemcomitans* enzyme dispersin B can disperse PIA-dependent staphylococcal biofilms by hydrolysis of the glycosidic linkages, which increases their susceptibility to antimicrobial treatment [195].

## BIOFILM REGULATION

The regulation of biofilms involves a complex network of regulatory cascades including QS-systems, regulatory small RNAs (sRNAs), alternative sigma factors, two-component systems and second messengers, such as c-di-GMP (**Fig. 1**). In this chapter we will highlight the most important regulatory circuits controlling biofilm formation in the pathogens *V. cholerae, P. aeruginosa, S. aureus* and *S. epidermidis.*

### Quorum sensing (QS)

QS is a bacterial system playing an important role in cell-to-cell communication and inter-kingdom signaling [196]. Bacteria produce extracellular signaling molecules, i.e. autoinducers, which accumulate with increasing cell density. After reaching a threshold concentration, autoinducers are recognized by receptors located on the cell membrane and orchestrate gene expression that underlie collective behaviors. This auto-regulation enables the bacteria to synchronize within its sessile microbial community in order to optimize adaption and resilience (e.g. luminescence, virulence, and biofilm formation) [197].

*V. cholerae* encodes four autoinducer receptors comprising the membrane-bound CqsS, LuxPQ, and CqsR as well as the cytoplasmic VpsS [198]. CqsA synthesizes the cholera autoinducer 1 (CAI-1), which binds to its cognate membrane-bound receptor CqsS, while LuxS produces the autoinducer 2 (AI-2) binding to the receptor LuxPQ [198]. Recent studies suggest that ethanolamine serves as a ligand for CqsR, while autophosphorylation of VpsS is blocked by nitric oxide via the nitric oxide-responsive hemoprotein NosP [199, 200]. Importantly, all four autoinducer receptors feed into the same phosphorelay pathway and converge at the histidine phosphotransfer protein LuxU [198]. Briefly, at low concentrations of autoinducers indicating low cell densities the kinase activity of the autoinducer receptors results in phosphorylation of LuxU, which in turn phosphorylates the transcriptional regulator LuxO [201]. Phosphorylated LuxO together with the alternative sigma factor RpoN activates transcription of the sRNAs Qrr1-4, which block the expression of HapR [201]. Notably, the transcription factor HapR is the main repressor of biofilm formation via transcriptional silencing of *vps* gene expression [201]. Moreover, HapR alters expression of several enzymes involved in biosynthesis of the second messenger c-di-GMP, which facilitates biofilm formation (see below). At high cell densities, the QS cascade decreases intracellular c-di-GMP levels via HapR [202]. Not surprisingly, *hapR* mutants show excessive exopolysaccharide production and uncontrolled biofilm formation [181]. Notably, several additional regulatory systems have been shown to influence the QS-HapR cascade, e.g. the two-component system VarA/S, the regulatory protein VqmA, cAMP and the alternative sigma factor RpoS, some of which will be discussed in the sections below [203–205]. In summary, QS in *V. cholerae* downregulates biofilm formation at high cell densities.

In *P. aeruginosa* three interconnected QS signaling networks have been discovered, i.e. the *las*-, the *rhl*-, and the PQS-system [206]. LasI produces the acylhomoserine lactone N-(3-oxododecanoyl)-homoserine lactone (OdDHL), which acts as autoinducer and binds to its cognate receptor LasR. Similarly, RhlI synthesizes the autoinducing molecule *N*-butyrylhomoserine lactone (BHL), which is recognized by RhlR. Notably, LasR and RhlR target genes constitute about 10% of the *P. aeruginosa* genome and thus both QS systems account for a majority of biofilm- and virulence-related processes in the pathogen [207]. PQS, structurally identified as 2-heptyl-3-hydroxy-4-quinolone, is synthesized by PhnAB and PqsABCDH from chorismate and was originally studied as an antibacterial compound [208]. Having a hydrophobic character PQS is transported via outer membrane vesicles [209]. Interestingly, PQS is a versatile compound. It not only activates genes involved in biofilm formation and virulence by binding to its receptor PqsR, but can also stimulate vesiculation by curvature induction of the outer membrane. Moreover, PQS chelates ferric iron and mediates iron acquisition. Undoubtedly, QS signaling networks of *P. aeruginosa* are interconnected in a hierarchical order (for an overview see [206]): LasR positively regulates the Rhl- and PQS-system, RhlR negatively regulates the PQS-system and PQS positively influences the RhL-system. QS signaling in *P. aeruginosa* impacts biofilm formation in multiple ways, e.g. via control of swarming and twitching motility, rhamnolipid biosynthesis, Psl expression, autolysis resulting in eDNA release and expression of the lectins LecA and LecB [206, 210–212].

In staphylococci, two regulatory QS systems have been described. The accessory gene regulator (Agr) system is considered to be the major QS regulator system in Gram-positive bacteria, while the LuxS system of staphylococci seems to play a minor role [213]. The *agr* locus contains two transcriptional units, RNAII and RNAIII, with their promotors P2 and P3, respectively [214]. The RNAII cluster consists of four genes, *agrB, agrD, agrC*, and *agrA* [214]. The peptide precursor for the extracellular QS AIP of the Agr system is encoded by *agrD* [215]. In most staphylococci, including *S. aureus* and *S. epidermidis*, the mature AIP contains a thiolactone modification between the central cysteine and the C-terminus of the seven to nine amino acid long AIP. This modification is added by the transmembrane endopeptidase AgrB, which additionally catalyzes the C-terminal cleavage and export of the AIP into the extracellular milieu [216]. After secretion AIP is finally trimmed by the type I signal peptidase SspB [217]. AIP activates the two-component signal transduction system AgrC/A, composed of the transmembrane histidine kinase sensor AgrC and its associated response regulator AgrA [218]. AIP signaling results in phosphorylated AgrA, which promotes transcription of the RNAII and RNAIII regions as well as the genes encoding the phenol-soluble modulins PSMα and PSMβ [219–221]. The latter represent surfactants required along the development of mature biofilms and detachment reducing the non-covalent interactions between cells and the biofilm matrix components [222, 223]. Regulation of RNAIII by AIP provides a positive autofeedback loop, as the RNAII cluster is responsible for AIP synthesis [214]. In contrast, RNAIII is a multifunctional regulatory RNA and represents the Agr intracellular effector molecule controlling expression of downstream targets [224]. Aside from virulence-associated factors, RNAIII regulates several biofilm-relevant factors like MSCRAMMs, nucleases, and peptidases [167, 225]. Notably, RNAIII also acts as a mRNA encoding for the δ-toxin (also known as δ-hemolysin) [226]. In general, the Agr QS system seems to have an inhibitory role on biofilm development as *agr* mutants exhibit thicker, less structured biofilms [227–229]. However, enhanced biofilm formation can be beneficial in clinical settings. Thus, mutants with impaired Agr signaling are frequently isolated from indwelling devices [227]. As in *V. cholerae*, the LuxS system uses the AI-2 autoinducer, suggesting a role in interspecies communication [230]. The LuxS system of *S. aureus* has been implicated in the regulation of biofilm formation, virulence, capsule synthesis, and antibiotic susceptibility [231–233]. Similarly, LuxS of *S. epidermidis* affects a number of genes, including biofilm exopolysaccharide biosynthesis gene clusters [234]. However, several reports argue that the observed phenotypes in mutants with an impaired LuxS system are due to its primary role in metabolism [234, 235]. Thus, LuxS as a QS regulatory system in staphylococci remains under debate.

### Cyclic-di-GMP

The second messenger bis-(3′-5′)-cyclic dimeric guanosine (c-di-GMP) is a central regulatory element mediating bacterial transitions between planktonic and sessile lifestyles. In general, c-di-GMP represses motility and virulence, but promotes biofilm formation, e.g. via activation of extracellular matrix production and adhesins [236]. Along biofilm development c-di-GMP can impact initial attachment, maturation, and detachment. Intracellular levels of c-di-GMP are controlled by two enzyme classes, diguanylate cyclases (DGCs) with their catalytical domain GGDEF, which synthesize c-di-GMP from GTP, and EAL- or HD-GYP-domain containing phosphodiesterases (PDEs), which hydrolyze c-di-GMP to 5′-phosphoguanylyl-(3′-5′)-guanosine or GMP, respectively [237]. Regulation of gene expression via c-di-GMP can be mediated at multiple levels, including (i) allosteric regulation of enzyme activity, (ii) binding and conformational change of transcription factors, and (iii) interactions with the untranslated regions of mRNAs, also known as riboswitches.

*V. cholerae* encodes for 31 GGDEF-domain, twelve EAL-domain, nine HD-GYP-domain containing proteins [238]. Moreover, ten additional proteins contain both, an GGDEF- and EAL-domain, but mostly exhibit only one activity as one domain is generally degenerated [238]. Transcriptional and post-transcriptional regulation of these proteins enable *V. cholerae* to modulate intracellular c-di-GMP levels. In addition to environmental signals, such as temperature, polyamines and bile salts, the transcriptional regulators of biofilm formation, i.e. HapR, VpsT and VpsR have been reported to regulate defined sets of PDEs and DGCs [203, 239–242]. VpsT and VpsR, which are central transcriptional response regulators of biofilm formation, induce DGCs and repress PDEs, thereby generating high c-di-GMP levels promoting biofilm formation [203, 240]. In contrast, HapR was shown to upregulate PDEs and downregulate DGCs, resulting in reduced c-di-GMP levels and less biofilm formation [202, 240]. Moreover, LuxO and the sRNAs Qrr1-4 also regulate some PDEs and DGCs in an HapR-independent manner [243]. Expression changes upon varying c-di-GMP levels are mediated by c-di-GMP-dependent riboswitches or c-di-GMP-binding proteins, e.g. transcriptional regulators FlrA, VpsR and VpsT as well as PilZ proteins [244–247]. PilZ-domain proteins represent a unique c-di-GMP binding protein family with an R*XXX*R and D*X*S*XX*G motif and are named after the type IV pilus control protein first identified in *P. aeruginosa* [248]. In *V. cholerae*, c-di-GMP represses transcription of flagellar genes by binding the flagellar regulatory protein FlrA, altering its activity, and thus inhibit its ability to activate flagellar gene expression [249]. VpsR binds c-di-GMP and upregulates the *vpsT*, the *vps*-I and -II gene clusters, encoding VPS synthesis and export, as well as the epsC-N operon, encoding the type II secretion machinery, required for secretion of biofilm matrix proteins, in a c-di-GMP-dependent manner [240, 250]. VpsT can undergo c-di-GMP-dependent and -independent dimerization, while c-di-GMP binding stabilizes the VpsT dimer [246]. VpsT facilitates expression of matrix proteins, i.e. RbmC and Bap1, and acts in concert with VpsR to activate the *vps* genes [240, 251, 252]. VpsR is also the main transcriptional regulator of the CraA adhesin [84]. Notably, mutual transcriptional activation of *vpsR* and *vpsT* also depends on c-di-GMP, antagonizing the H-NS repression of *vps* and *rbm* genes [253]. Additionally*, V. cholerae* expresses five PilZ-domain proteins, which affect virulence, motility, and biofilm formation [244]. However, the molecular mechanism how c-di-GMP sensing by PilZ proteins is transferred to changes in the transcriptional profile remains to be elucidated.

The second messenger c-di-GMP also promotes biofilm formation in *P. aeruginosa*. Biofilms of this pathogen contain on average 75-110 pmol c-di-GMP per mg of total cell extract, which is approximately four-fold higher compared to planktonic cells [254]. Similar to *V. cholerae, P. aeruginosa* encodes a diverse set of DGCs and PDEs including 18 GGDEF-domain-, five EAL-domain, three HD-GYP-domain-containing proteins as well as 16 proteins with GGDEF- and EAL-domains [255]. Along the latter, “hybrid” proteins with DGC and PDE activity exist in *P. aeruginosa* [256]. For example, MucR exhibits DGC activity in planktonic cells promoting alginate biosynthesis, whereas in biofilms PDE activity dominates resulting in nitric oxide- or glutamate- induced biofilm dispersal [257]. The first characterized DGC of *P. aeruginosa* is WspR, which is named after the wrinkly spreader phenotype forming upon an increased exopolysaccharide production [88]. WspR activity is controlled by three subsequent events: phosphorylation via the chemosensor WspA upon surface contact, which activates c-di-GMP synthesis [88, 258], followed by oligomerization and cluster formation, which increases enzyme activity [259], and finally, c-di-GMP-dependent feedback inhibition [260]. Contrarily, RocR is an example for a PDE of *P. aeruginosa*, representing the response regulator in the RocSAR signaling system, which is composed of a membrane sensor RocS1 and two response regulators RocR and RocA1 [227, 261]. The Roc system regulates biofilm formation and virulence gene expression, i.e. the *cup* fimbriae gene clusters or type III secretion system genes [261, 262]. Besides WspR and RocR, several other DGCs and PDEs have been reported to play a role in *P. aeruginosa* biofilm formation. In addition to WspR at least four other DGCs, i.e. SadC, RoeA, SiaD, and YfiN/TpbB, control the transition from the planktonic to the biofilm lifestyle [263–265], while the two DGCs GcbA and NicD as well as the three PDEs DipA, RbdA and NbdA are involved in dispersal of mature biofilms [266–270]. Currently, more than a dozen c-di-GMP-recognizing effectors have been identified in *P. aeruginosa* with about half of them harbouring a PilZ domain [255]. One of them is the membrane-associated protein Alg44, which is required for alginate production [271]. The transcription factor FleQ activates flagellar gene expression and represses *pel, psl* and *cdr* genes upon low levels of c-di-GMP [272]. However, when c-di-GMP levels increase FleQ changes into an activator, even though FleQ lacks a PilZ domain [273]. Notably, c-di-GMP not only binds to DGCs, PDEs, PilZ domains and transcriptional factors, but may also act as a competitive inhibitor for ATP catabolizing enzymes, such as the FliI flagellar ATPase [274].

There is also growing evidence for c-di-GMP signaling in Gram-positive bacteria, predominantly studied in *Bacillus subtilis, Clostridium difficile* and *Listeria monocytogenes* [275]. Impact of c-di-GMP on staphylococci is under debate. Although *S. aureus* and *S. epidermidis* cannot metabolize c-di-GMP they encode the degenerated GGDEF-protein CdgS, which lacks cyclase activity [276, 277]. Notably, deletion of *gdpS* reduces biofilm development in both pathogens, however, whether GdpS senses c-di-GMP is controversial [276, 277].

### Regulatory small RNAs (sRNAs)

sRNAs have diverse roles as auxiliary regulators affecting QS in a direct or indirect way. This involves mediating the amount of autoinducer synthesis, integrating different regulation inputs or regulating crosstalk among different network components.

The four Hfq-dependent quorum regulatory RNAs (Qrr1-4) of *V. cholerae* prevent translation of the *hapR* mRNA by base pairing to the 5′-untranslated region (UTR) [278]. Along the QS signaling cascade low autoinducer concentrations result in phosphorylated LuxO, which together with the alternative sigma factor σ^5^ induces Qrr sRNA expression [279]. The four Qrr sRNAs of *V. cholerae* compensate each other and thus can all fully inhibit HapR [280]. On the other side, Qrrs promote *aphA* mRNA translation by revealing the ribosome binding site [281]. AphA is not only a transcriptional activator of virulence, but also of biofilm formation by promoting expression of the biofilm regulator VpsT [282]. Recently, an RNA-seq study identified the sRNA VqmR to inhibit biofilm formation by translational silencing of the *vpsT* mRNA [283]. Moreover, the sRNAs CsrB, CsrC and CsrD, which are controlled by the two-component system VarS/A, block activity of the RNA-binding CsrA [284]. The carbon storage regulatory protein CsrA is a major regulator for carbon storage, but also impacts biofilm formation and virulence [284, 285]. CsrA can impact the QS cascades by interfering in LuxO-dependent activation of the sRNAs Qrr1-4 resulting in elevated HapR expression as well as stimulation of HapR activity [284, 286].

In *P. aeruginosa* the CsrA-homolog RsmA (regulator of stationary phase metabolites) is controlled by four sRNAs, i.e. RsmZ, RsmY, RsmW and RsmV [287–290]. The primary sRNAs that sequester RsmA are RsmZ and RsmY, while RsmV and RsmW seem to play accessory roles. RsmA acts as a negative post-transcriptional regulator of biofilm formation blocking Psl exopolysaccharide production and other QS-controlled genes [291, 292]. The two sRNAs RsmZ and RsmY are positively controlled by the LadS-RetS-GacS/A signal transduction pathway [291, 293, 294]. Expression of RsmV and especially RsmW rises with increasing cell density [287, 290]. Recently, the RNA-binding protein RsmF, a structurally distinct homologue of RsmA, has also shown to bind sRNAs and thereby reduce biofilm formation capacity [289, 295]. Another *P. aeruginosa* sRNA negatively regulating biofilm formation is CrcZ, which is upregulated under anaerobic conditions [296]. Originally identified as a decoy that sequesters Hfq during relief of carbon catabolite repression, the exact physiological role of CrcZ along biofilm formation remains to be elucidated [297]. Moreover, PQS synthesis in *P. aeruginosa* is stimulated by the sRNA PhrS [298]. PhrS is highly expressed under oxygen limiting conditions as it is positively controlled by the oxygen-responsive regulator ANR [298].

More than 250 sRNAs have been discovered in *S. aureus*, but functional studies are still lacking for most of them [299]. An extensively studied regulatory RNA is the 514 nucleotide-long RNAIII representing the multifunctional effector of the Agr QS cascade. Interestingly, RNAIII contains a small open reading frame encoding the δ-hemolysin, but also functions as a regulatory sRNA controlling translation initiation and mRNA stability of a number of *S. aureus* transcripts via base-pairing with their 5′-UTR [224, 226]. Unlike many other sRNAs, RNAIII is Hfq-independent, but requires the RNA-binding protein strand-specific endoribonuclease III (RNase III), which degrades several RNAIII-targeted mRNAs [300]. Besides others, RNAIII negatively regulates translation of the transcription factor Rot and the cell surface protein A, while translation of the transcription factor MgrA is promoted [301]. Overall, this reduces biofilm formation as the surface protein Spa acts as adhesin, Rot represses several secreted proteases capable of biofilm matrix degradation, and MgrA downregulates expression of the phenol-soluble modulins important along biofilm formation [302, 303]. The *icaR* mRNA, encoding the transcriptional repressor of PIA synthesis, exhibits a negative feedback regulation as the 3′-UTR interferes with translation of its own RNA [304]. Moreover, the 5′-UTR of *sarA* contains the sRNA teg49, which facilitates expression of the transcriptional regulator SarA and thereby promotes biofilm formation [305].

### Transcriptional regulators, two-component systems, and alternative sigma factors

VpsR and VpsT represent the two major positive regulators of biofilm formation in *V. cholerae*, which control expression of the VPS exopolysaccharide and biofilm matrix proteins [240]. Complexity of the regulatory network is given by their overlapping, but not identical function and by the fact that they activate each other's expression [251]. While VpsT activity is independent of phosphorylation, VpsR activation requires phosphorylation on the D59 amino acid residue [81]. However, a sensor histidine kinase catalyzing the phosphotransfer has not yet been identified. VpsR and VpsT are repressed by the QS regulator HapR, while VpsT is under positive control of AphA and stringent response in a RpoS-dependent manner [240, 282, 306]. In addition to the QS system, activity of HapR is also modulated by the two-component system VarS/VarA via the sRNAs *csrB-D* and their binding protein CsrA (see above) [284, 286]. The signal sensed by VarS remains to be elucidated. Moreover, HapR expression is activated by the transcriptional regulator VqmA at low cell density and by the alternative sigma factor RpoS [203, 204].

In *P. aeruginosa* two-component systems aid in a stage-specific control of biofilm formation, e.g. the GacA/S, BfiR/S, BfmR/S, and MifR/S regulatory networks [307]. The GacA/S two-component system controls the sRNAs RsmZ and RsmY, which act as antagonists of RsmA modulating Psl exopolysaccharide production (see sRNA section for details) [294, 308]. Phosphorylation of GacA via GacS is inversely regulated by RetS and LadS. While RetS interferes in GacS autophosphorylation by formation of RetS/GacS heterodimers, LadS stimulates GacA phosphorylation [294, 309]. The BfiR/S, BfmR/S, and MifR/S systems regulate defined steps along the *P. aeruginosa* biofilm formation. BfiR/S is involved in the transition into the biofilm stage and initial attachment, BfmR/S controls biofilm maturation after irreversible attachment, and MifR/S is rather relevant for subsequent microcolony formation [307]. The alternative sigma factor AlgU (also known as RpoE) is responsible for transcription of the alginate biosynthetic operon and a key factor along the conversion from the non-mucoid to the mucoid phenotype in CF [310]. This conversion is frequently based on spontaneous mutations in *mucA*, encoding the anti-sigma factor of AlgU [311]. This results in increased activity of AlgU and consequently alginate overproduction [311, 312].

Most *S. aureus* strains encode 16 different two-component systems [313]. Among them, the oxygen-dependent system SrrAB, has been shown to regulate pathogenicity, promoting PIA biosynthesis upon activation of *icaADBC* transcription via IcaR and, thus facilitating biofilm formation [314, 315]. A major transcriptional regulator relevant for biofilm formation is the staphylococcal accessory regulator SarA [316]. SarA positively regulates the expression of Agr and RNAIII, but also regulates several genes involved in biofilm formation directly [317]. Representative examples include the fibronectin-binding proteins FnbPA and FnBPB as well as the cell wall adhesin Bap, which are activated, while nucleases and proteases are repressed [316, 318]. The stress-induced alternative sigma factor SigB is also crucial for biofilm formation in *S. aureus* and *S. epidermidis* [253, 319]. In *S. aureus sigB* mutants are impaired for *in vitro* biofilm formation, most likely due to an unleashed activation of the Agr system and massive upregulation of proteases and nucleases [129, 188, 190, 253]. Similarly, *sigB* mutants show reduced biofilm formation in *S. epidermidis*, which correlates with lower PIA production [319, 320].

## THERAPEUTICAL INTERVENTION STRATEGIES AGAINST BACTERIAL BIOFILMS

As discussed above, biofilms are a troublesome barrier to components of the host immune system as well as many treatments conventionally used to solve microbial infections, especially antibiotics. Due to the medical importance of bacterial biofilms, effective therapeutical strategies targeting biofilms are highly relevant for clinical applications. Indeed, several potential clinical interventions for treatment of bacterial biofilms associated with infections have been recently suggested.

Despite having lower effectiveness, conventional antibiotics can be used to treat biofilms to some extent. However, antibiotic therapy needs to be evaluated case-by-case, since the efficacy varies depending on the bacterial species, location of the infection, and mode of delivery. Otherwise, ineffective use of antibiotics increases the risk of antibiotic resistance [321]. Antibiotic lock therapy, a technique based on coating the lumen of catheters with a small volume of a concentrated antimicrobial cocktail, has been used for many years as a preventive measure against bacterial adhesion to catheter surfaces [322]. In the case of CF-associated chronic *P. aeruginosa* lung infection, the use of aerosolized antibiotic mixtures has positive results when compared to oral or intravenous administration, and many commercial aerosol formulations are available [323]. Furthermore, novel compounds such as antimicrobial peptides and peptide nucleic acids are highly effective at preventing biofilm formation and breaking down mature biofilms, thus showing great promise for future human use [324, 325].

Among the identified regulatory pathways involved in biofilm formation, QS is not only the most important regulatory circuit, but also found in many bacterial species. Consequently, disruption of QS, named quorum quenching, can be a promising medical application to treat biofilm-associated infections. Using different chemicals, quorum quenching can be performed at several levels, namely preventing bacterial adhesion, inhibiting biofilm maturation, or causing mature biofilms to disintegrate. Although quorum quenching does not kill bacteria, it renders them more sensitive to conventional therapies and could be used in combination with antibiotics, for instance. Several approaches of this kind have been reported successful in *P. aeruginosa* and *S. aureus* biofilms [326]. As highlighted throughout this review, c-di-GMP is an important signaling molecule promoting biofilm formation in many bacteria [327]. Inhibitors of c-di-GMP-synthesizing diguanylate cyclases have recently been discovered and already shown to effectively inhibit biofilm synthesis in *P. aeruginosa* and *A. baumanii* [328].

Nowadays the chemical composition of the biofilm matrix is known for most pathogenic microbes, and so, a viable option would be to disperse biofilm-enclosed bacterial cells by degrading the matrix. One major component of many bacterial biofilms is eDNA, and bacteria produce their own nucleases to digest the eDNA for, among other ends, dispersing the biofilm matrix depending on environmental conditions [329]. Many strategies were employed in previous studies, such as artificial upregulation of bacterial nucleases and treatment of biofilms with exogenous DNase, which were successful in dispersing biofilms [329]. As such, nucleases have potential to become therapeutic agents in a similar way to quorum quenching agents, destroying the protective matrix, and rendering bacteria sensitive to other treatments. Clinical *P. aeruginosa* isolates tend to produce high levels of the biofilm matrix component alginate, an indicator for serious disease prognoses in CF patients [312]. Alginate lyases, which degrade alginate polymers, have been extracted and purified from several bacterial species and successfully used in combination with antibiotics to treat clinical *P. aeruginosa* biofilms [330]. Moreover, many members of the *Enterobacteriaceae* family produce extracellular amyloid fibers, which are detrimental for their ability to adhere to surfaces and to generate and maintain biofilms. In this context, specific bioactive compounds have been identified, which inhibit formation of these fibers, and which effectively prevent biofilm formation and destabilize mature biofilms in pathogenic *E. coli* [331].

The impact of LecA and LecB on *P. aeruginosa* virulence and biofilm formation initiated therapeutical strategies based on compounds specifically inhibiting binding capacities of these lectins [156]. Beneficial effects were observed upon co-administration of lectin-inhibiting carbohydrates in a lung infection animal model, highlighted by reduced lung injury and mortality compared to the control group [156].

Weak acids, namely the acetic acid present in vinegar, have been used empirically as disinfectants for thousands of years, but research into their antibacterial effects only started to flourish in the last century [332]. Presently, *in vitro* and *in vivo* assays have proven that weak organic acids, as well as derivative drugs and salts, are extremely effective in penetrating the biofilm matrix and killing biofilm-implanted *P. aeruginosa* and *S. aureus*, as well as planktonic cells [333]. Especially, weak acids show strong potential as a topical treatment for wound biofilms [333].

Use of bacteriophages is considered an advantageous alternative to antibiotics in the treatment of antibiotic-resistant infections [334]. The same strategy could be applied to biofilm-associated infections, since it is known that some phages possess hydrolytic enzymes on their surface, enabling them to invade the biofilm matrix and infect bacteria inside biofilms [335]. For example, degradation of the alginate polymer has been reported for *P. aeruginosa* phages [336]. Furthermore, lysogenic phages can be genetically engineered, providing useful and versatile tools for not only inducing lysis in biofilm-rooted cells, but also modulating their behavior in many other ways [337]. Phage therapy has proven effective in ameliorating *P. aeruginosa* biofilm infections in chronic otitis patients and in a murine chronic lung infection model [338, 339]. Bacteriophage treatment combined with prior debridement of biofilm material significantly improved wound healing in a chronic *S. aureus* wound infection model [340]. However, there are still limitations to the usage of phages such as the risk of development of bacterial resistance against phages, the possibility of undesired horizontal gene transfer via lysogenic phages to share virulence-related genetic elements across the biofilm community, and phage immunogenicity resulting in generation of neutralizing antibodies by the human host, which could translate into inflammatory side effects [337].

## CONCLUDING REMARKS

Bacterial biofilms pose a great challenge to our health care system as they are involved in various human diseases and exhibit high antimicrobial resistance. Clinical relevance ranges from biofilms formed on diverse body surfaces, medical devices-related biofilms, and environmental biofilms of facultative pathogens, which act as a reservoir for infections. Given the global burden of biofilm-associated infections, therapeutic intervention strategies targeting biofilms are desperately needed. Using selected model organisms, we have made substantial progress to understand biofilm development, composition and regulation. This revealed common principles as well as species-specific differences, which resulted in new therapeutic strategies to promote biofilm dispersal or even combat initial biofilm formation. However, future initiatives need to translate *in vitro* results of promising therapeutic agents to *in vivo* systems and clinical trials. This might be hampered by increasing evidence that *in vitro* biofilm formation under laboratory settings not always reflects the *in vivo* situation. Relevant differences between *in vitro* and *in vivo* biofilm formation include chemical composition of the abiotic surfaces, presence of host-derived factors or interactions with other microbes resulting in multi-species biofilms. It will be a challenging, but necessary task for future research to develop new biofilm models that more closely reflect the *in vivo* situation.

## Abbreviations:

AIP: – autoinducing peptide;
CF: – cystic fibrosis;
COPD: – chronic obstructive pulmonary disease;
DGC: – diguanylate cyclase;
eDNA: – extracellular DNA;
EHEC: – enterohemorrhagic E. coli;
EPS: – extracellular polymeric substance;
GlcNAc: – N-acetyl-D-glucosamine;
IBD: – inflammatory bowel disease;
MRSA: – methicillin-resistant S. aureus;
MSCRAMM: – microbial surface components recognizing adhesive matrix molecule;
MSHA: – mannose sensitive hemagglutinin;
PIA: – polysaccharide intercellular adhesin;
PDE: – phosphodiesterase;
QS: – quorum sensing;
SERAM: – secretable expanded repertoire adhesive molecule;
sRNA: – small RNA;
UPEC: – uropathogenic E. coli;
UTI: – urinary tract infection;
UTR: – untranslated region;
VPS: – Vibrio exopolysaccharide.
